# Comments on the NEMA NU 4-2008 Standard on Performance Measurement of Small Animal Positron Emission Tomographs

**DOI:** 10.1186/s40658-020-0279-2

**Published:** 2020-02-24

**Authors:** Patrick Hallen, David Schug, Volkmar Schulz

**Affiliations:** 1 Department of Physics of Molecular Imaging Systems, Institute for Experimental Molecular Imaging, RWTH Aachen University, Pauwelstraße 19, Aachen, 52074 Germany; 2 Hyperion Hybrid Imaging Systems GmbH, Pauwelstraße 19, Aachen, 52074 Germany; 3 III. Physikalisches Institut B, RWTH Aachen University, Otto-Blumenthal-Straße, Aachen, 52074 Germany; 4 Fraunhofer Institute for Digital Medicine MEVIS, Forckenbeckstrasse 55, Aachen, 52074 Germany

**Keywords:** Positron emission tomography, Small-animal imaging, Performance evaluation

## Abstract

The National Electrical Manufacturers Association’s (NEMA) NU 4-2008 standard specifies methodology for evaluating the performance of small-animal PET scanners. The standard’s goal is to enable comparison of different PET scanners over a wide range of technologies and geometries used. In this work, we discuss if the NEMA standard meets these goals and we point out potential flaws and improvements to the standard.For the evaluation of spatial resolution, the NEMA standard mandates the use of filtered backprojection reconstruction. This reconstruction method can introduce star-like artifacts for detectors with an anisotropic spatial resolution, usually caused by parallax error. These artifacts can then cause a strong dependence of the resulting spatial resolution on the size of the projection window in image space, whose size is not fully specified in the NEMA standard. If the PET ring has detectors which are perpendicular to a Cartesian axis, then the resolution along this axis will typically improve with larger projection windows.We show that the standard’s equations for the estimation of the random rate for PET systems with intrinsic radioactivity are circular and not satisfiable. However, a modified version can still be used to determine an approximation of the random rates under the assumption of negligible random rates for small activities and a constant scatter fraction. We compare the resulting estimated random rates to random rates obtained using a delayed coincidence window and two methods based on the singles rates. While these methods give similar estimates, the estimation method based on the NEMA equations overestimates the random rates.In the NEMA standard’s protocol for the evaluation of the sensitivity, the standard specifies to axially step a point source through the scanner and to take a different scan for each source position. Later, in the data analysis section, the standard does not specify clearly how the different scans have to be incorporated into the analysis, which can lead to unclear interpretations of publicized results.The standard’s definition of the recovery coefficients in the image quality phantom includes the maximum activity in a region of interest, which causes a positive correlation of noise and recovery coefficients. This leads to an unintended trade-off between desired uniformity, which is negatively correlated with variance (i.e., noise), and recovery.With this work, we want to start a discussion on possible improvements in a next version of the NEMA NU-4 standard.

## Introduction

The National Electrical Manufacturers Association’s (NEMA) NU 4-2008 standard on “Performance Measurements of Small Animal Positron Emission Tomography” specifies “standardized methodology for evaluating the performance of positron emission tomographs (PET) designed for animal imaging” [1]. The standard’s goal is to enable comparison of the performance of different PET systems over a wide range of technologies and geometries used. Thus, the methods specified in the standard should not artificially favor or disfavor certain choices in scanner geometry and technology and the performance results should indicate the expected performance in real-world applications as closely as possible. Virtually all commercial small-animal PET systems and most research prototype PET systems have published performance evaluations based on the NEMA standard and Goertzen et al. [2] have published a review comparing small-animal PET systems based on the respective NEMA performance publications. These publications are an essential benchmark in the development of new PET systems and an important tool for the purchase decisions of potential buyers.

The NEMA standard specifies 5 measurements with respective analysis: evaluation of spatial resolution; evaluation of total, true, scattered, random, and noise-equivalent count rates; evaluation of system sensitivity; and quantitative evaluation of image quality in a standardized imaging situation using a hot-rod phantom.

The standard was devised over 10 years ago, so it does not incorporate newer technological developments and paradigm shifts. For instance, the use of data acquisition into sinograms and filtered backprojection reconstruction mandated in the standard was more widespread than it is today. Nowadays, these methods are often only implemented to evaluate the PET performance based on NEMA but never actually used for real-world applications

In this work, we examine if the NEMA standard meets its goals to enable a fair comparison of PET systems and we point out potential flaws and improvements in the standard. In our opinion, the standard is underspecified in several parts, limiting the comparability of different systems, since the investigators performing the performance evaluations are still free to choose parameters which significantly influence the results. The methods specified for evaluation of the spatial resolution disadvantages certain system geometries, where those geometries do not exhibit the same reduction in spatial resolution in real-world applications. The definition of random rates is circular and allows the use of very different other methods generating different results. The chapter on sensitivity is ambiguous, leading to publications using different or even unclear methods for the measurement of sensitivity, creating ambiguity in the interpretation of sensitivity of different PET systems.

If applicable, we demonstrate the claimed issues with simple simulation studies. All discussions in this work should be universally applicable to any PET system. However, it is still helpful and instructive to support the claims in this work with real-world data. This is done using data obtained with the Hyperion II ^D^ PET/MRI scanner, which was developed by our group [3]. Using the same data, we already have published a performance evaluation based on the NEMA standard [4].

The goal of this work is to start a discussion on a revised version of the NEMA standard and to provide input for this discussion.

## Spatial resolution

To evaluate the spatial resolution, the NEMA standard mandates the use of point source scans which are reconstructed using filtered backprojection. However, basically all modern PET scanners instead use an iterative maximum likelihood expectation maximization (MLEM) algorithm for reconstruction [4–15], so a scanner’s spatial resolution using filtered backprojection is not necessarily indicative of its spatial resolution for applications. While the mandated filtered backprojection is intended to benchmark the detector performance alone, we will demonstrate in the following that it disadvantages certain scanner geometries. Furthermore, the NEMA standard specifies that the spatial resolution must be determined using the projections of the reconstructed point sources inside a window in image space, without strictly specifying the size of this projection window. We will demonstrate that this can lead to an ambiguous spatial resolution which depends on the size of the projection window and allows for artificially enhancing the spatial resolution by choosing a particularly large projection window for certain scanner geometries.

**Fig. 1:**
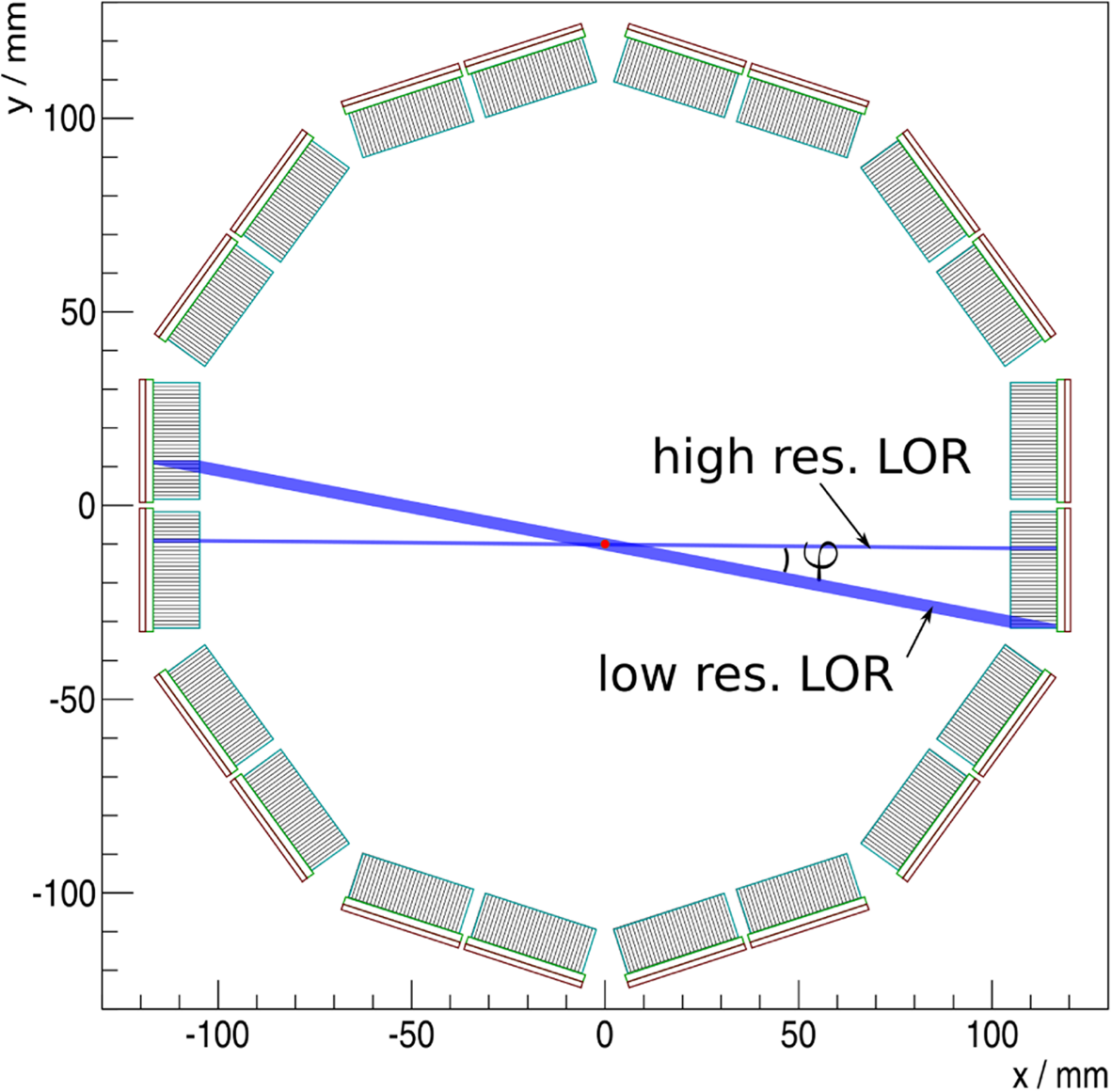
Ring geometry that was used for the simulations and the measurement. The blue bands show the parallax error of LORs, which increases approximately proportional to the angle *φ* to the normal of the block detector

The main disadvantage of filtered backprojection is that it does not include any model of the detector and assumes an ideal, ring-like PET scanner, while the detectors in real-world PET scanners are usually in a block geometry with anisotropic spatial resolutions. Line of responses (LORs) perpendicular to the detector’s front face are detected with the highest resolution, while tilted LORs have a parallax error in the detected position, which increases with more tilt of the LORs relative to the detector’s front faces as illustrated in Fig. 1. In principle, this effect can be reduced by detectors which are able to determine the depth of interaction (DOI) of the gamma interaction, but in practice most PET system do not employ detectors with DOI determination [4–6, 11, 12, 14, 16]. Additionally, PET rings have gaps between the detector, where no LORs are detected at all.

These issues with filtered backprojection will lead to artifacts in the reconstructed activity. For instance, each angle where the PET ring has an enhanced spatial resolution creates an excess in reconstructed activity along the line connecting this position with the point source and each angle with degraded spatial resolution creates a reduction in reconstructed activity along the respective line. Similarly, gaps between the detector create a lack of reconstructed activity along these lines.

**Fig. 2:**
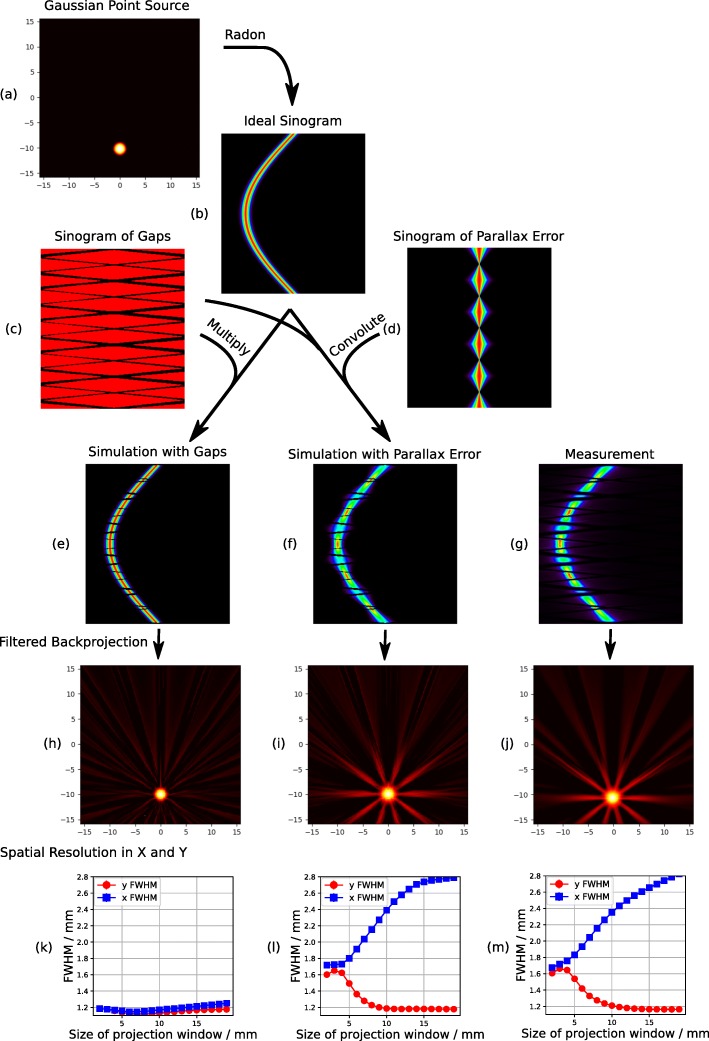
Visualization of the influence of anisotropic detector resolution on the filtered backprojection and resulting spatial resolution along the two axis. **e**, **h**, **k** The simulation with only gaps. **f**, **i**, **l** The simulation with anisotropic detector resolution and gaps of 10 detector modules. **g**, **j**, **m** A measurement. The simulation with anisotropic detector resolution and the measurement exhibit a star-like artifact in the reconstruction, which leads to a split in spatial resolution along *x* and *y* axis, as shown in the bottom row

To understand and demonstrate this behavior, it is instructive to look at these effects in sinogram space. In sinogram space, the enhanced spatial resolution of perpendicular LORs manifests as hot spots or rather peaks in the center of each detector modules as Fig. 2g shows. With increasing distance from the center of the detector module the spatial resolution degrades, blurring the line of the point source in sinogram space. We model this as the convolution of the sinogram of a Gaussian point source and the parallax error of the detector. The parallax error of the detector stack can be modeled as the shape of two triangles, connected at their tips as shown in Fig. 2d. The parallax error is proportional to sin*φ*, where *φ* is the angle to the normal of the block detector as defined in Fig. 1. The parallax error shown in Fig. 2d is a small-angle approximation of this.

In addition to the inherent problems of mandating the use of filtered backprojection in the NEMA standard, the standard additionally mandates projecting the reconstructed three-dimensional activity onto different one-dimensional axes using a projection window. However, the size of the projection window is not fully specified: “The response function is formed by summing all one-dimensional profiles that are parallel to the direction of measurement and *within at least two times* the FWHM of the orthogonal direction” [1, p. 7]. The first issue is that this definition is circular, since the minimal size of the projection window to determine the FWHM is defined using the FWHM itself. One can easily fix this problem, either using a sufficiently large projection window in the first place, or by reducing the size of the projection window iteratively in dependence of the determined FWHM in the previous iteration. However, the much bigger problem is that the size of the projection window can strongly influence the resulting spatial resolution. The cause of this is the integration of the star-like artifacts created by the anisotropic spatial resolution, as we demonstrate with the following simulation, shown in Fig. 2.

We created the activity distribution of an ideally reconstructed point source by assuming a rotationally symmetric two-dimensional normal distribution, shown in Fig. 2a. The position of the point source is off-center at a radial offset of 10 mm. To investigate the essence of the effects, we do not include noise in our simulation. From this ideally reconstructed point source, we create a sinogram by forward projection (i.e., by applying a Radon transformation). The resulting sinogram is shown in Fig. 2b.

We include the gaps between the detector stacks in our simulation by creating a sensitivity sinogram, where all bins corresponding to gaps are 0 and bins corresponding to sensitive detector area are 1 shown in Fig. 2c. The simulated geometry is depicted in Fig. 1 and follows the geometry of the Hyperion II ^D^ scanner to allow a comparison between simulation and measurement. When we include this model of gaps in our simulation by multiplying the sensitivity sinogram with our point-source sinogram (Fig. 2e) and then performing a filtered backprojection (i.e., an inverse Radon transformation), we get a reconstructed point source with slight artifacts, shown in Fig. 2h. As stated above, the artifacts are a lack of reconstructed activity along the lines connecting the gaps and the point source. When analyzing the spatial resolution of the filtered backprojection with gaps, we observe little influence of the gaps compared to the filtered backprojection of an ideal sinogram without gaps. More importantly, the resulting spatial resolution of 1.2 mm FWHM is stable to changes in the size of the projection window, as shown in Fig. 2k. Thus, gaps between the detectors are not the cause of severe artifacts and only have a very minor influence on the resulting spatial resolution with the usually small gaps of PET scanners.

When we additionally include the effect of the anisotropic detector resolution due to parallax errors by convolving the point-source sinogram and the point spread function in Fig. 2d, the resulting filtered backprojection in Fig. 2i exhibits a star-like artifact, i.e., the lines connecting the center of each detector stack and the point source exhibit a visible excess in activity.

**Fig. 3:**
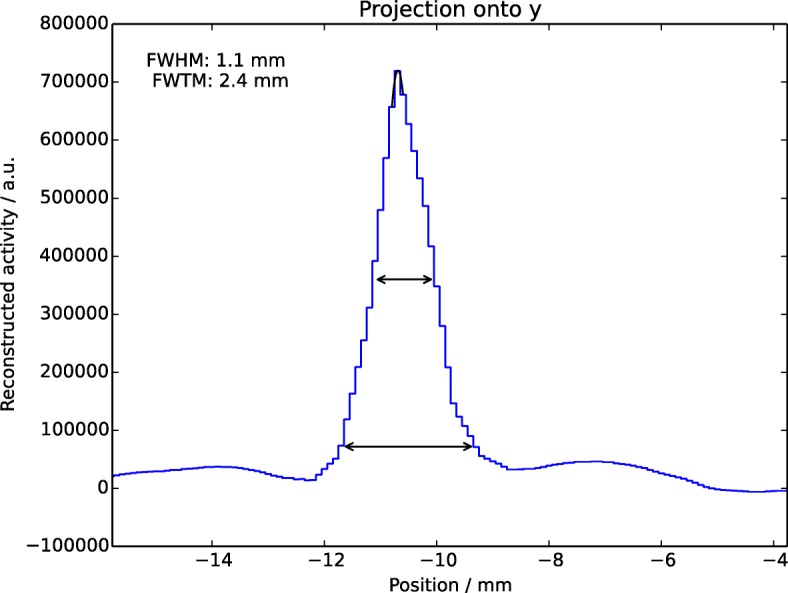
Line profile of the reconstructed point source projected onto the *y* axis. The star-like artifact which is aligned with the *x* axis creates an excess in activity at the peak of the profile boosting the spatial resolution

If one of these excesses aligns with one of the Cartesian projection axis, and with the simulated geometry they do so for the *x* axis, the projection onto the axis perpendicular to this axis will result in a peaked excess at the maximum of the line profile, as shown in Fig. 3. A scanner’s spatial resolution is defined by the FWHM and FWTM of this profile, which depends strongly on the height of the maximum. Therefore, a peaked excess of the maximum will significantly enhance the resulting spatial resolution. For our geometry, this enhancement is only observed for the *y* axis, because only the *x* axis has an excess in activity aligned with it, as there are not any detector stack which are perpendicular to the *y* axis. This difference between the resolution in *x* and *y* is essentially an artifact and basically non-existent in real-world applications using an iterative maximum likelihood expectation maximization (MLEM) reconstruction. More importantly, the extent of this effect depends strongly on the size of the projection window as demonstrated in Fig. 2k. Increasing the size of the projection window enhances the resulting spatial resolution in *y* (i.e., decreases FWHM and FWTM) while degrading the spatial resolution in *x*. This makes comparison of the spatial resolution of different PET system difficult and maybe even impossible, as the NEMA standard does neither specify a clear projection window size nor does it mandate that the used window size should be reported. Thus, most publications do not state the used projection window [5, 7, 14, 16]. Other geometries may not exhibit this behavior at all, favoring or disfavoring systems which have detectors perpendicular to a Cartesian axis.

The measurement and filtered backprojection reconstruction of point sources with the Hyperion II ^D^ scanner shown in Fig. 2g and j look very similar to the simulation which includes parallax error and gaps: The sinogram has the same hot spots at the angles where the line of responses is perpendicular to the detector surface and the reconstruction exhibits the same star-like artifact. The analysis of the reconstruction yields the same observed difference in spatial resolution between the *x* and *y* axis. Additionally, we observe the same strong dependence on the size of the projection window, shown in Fig. 2m.

An extreme example of a scanner geometry affected by this issue would be a box geometry instead of the conventional ring geometry, i.e., a PET scanner with 4 large perpendicular detector modules without DOI capabilities. With such a geometry, the filtered backprojection artifact would have the shape of a cross, with both lines of excessive activity aligned with the *x* and *y* axis. Thus, the artifact would enhance the resolution along both *x* and *y* axis by boosting the maximum of both projections. This scenario is not solely hypothetical, as small-animal PET scanners with the described box-like geometry exist such as PETbox 4 [17]. In PETbox’s NEMA NU-4 performance evaluation they state that using FBP was not possible “since a FBP algorithm specific for the PETbox4 system with the unconventional geometry has not been developed” [17, p. 3797].

Other examples of published performance evaluation which have omitted the filtered backprojection altogether when evaluating the spatial resolution are [8, 18]. This is an indication that these groups do not find the results based on filtered backprojection not indicative for the performance of their system.

Fixing the issues of this method and proposing a better method to evaluate the spatial resolution is challenging. The NEMA standards committee surely knew many of these issues and we believe most of the PET community will be aware of issues with filtered backprojection, as well. However, so far, none of the performance publications based on NEMA discussed the issues presented here, so we believe it is worthwhile to state them to start a discussion.

One obvious solution would be to simply not use filtered backprojection and to perform the reconstruction with the default reconstruction method provided with the scanner, which is also used for the evaluation of the image quality phantom and for real-world applications. In modern scanners, this is usually an iterative reconstruction algorithm, e.g., ordered subset expectation maximization [19] and maximum likelihood expectation maximization [20, 21]. However, these algorithms can artificially enhance the spatial resolution of point sources without background activity due to, e.g., the non-positivity constraint or resolution recovery [22–24]. Thus, the reconstruction of a point source would mostly be a benchmark of the reconstruction and not of the underlying detector performance. We suspect that these arguments were the main reason why the NEMA standards committee chose filtered backprojection instead.

One alternative could be the evaluation of spatial resolution using a Derenzo hot-rod phantom. The standard could specify the geometry of such a phantom, specify the activity and scan time, allow the use of the reconstruction method supplied by the manufacturer, and then define a quantitative analysis method. The Derenzo phantom is already well-established in the community as a benchmark to evaluate the spatial resolution. For instance, several NEMA performance publications already include such a measurement as a benchmark of spatial resolution [5, 7, 12, 15]. However, these results are not easily comparable, as there currently is no standardized quantitative analysis method to determine the spatial resolution from a measurement of a Derenzo phantom. Usually, the spatial resolution is estimated by making a qualitative judgment at which distance the hot rods are still discernible. In principle, such a definition of spatial resolution based on the ability to resolve to close points is very reasonable and commonly used as a definition of spatial or angular resolution for telescopes and microscopes [25, 26]. However, for a quantitative definition of spatial resolution, there must be a standardized limit of the peak-to-valley ratio between two resolvable point sources, i.e., how much the intensity between the two peaks must dip to make them just resolvable. In a new standardized definition of PET spatial resolution, the PET community could follow the commonly used Rayleigh criterion with an intensity dip of 26.5% [27], or standardize a different limit.

**Fig. 4:**
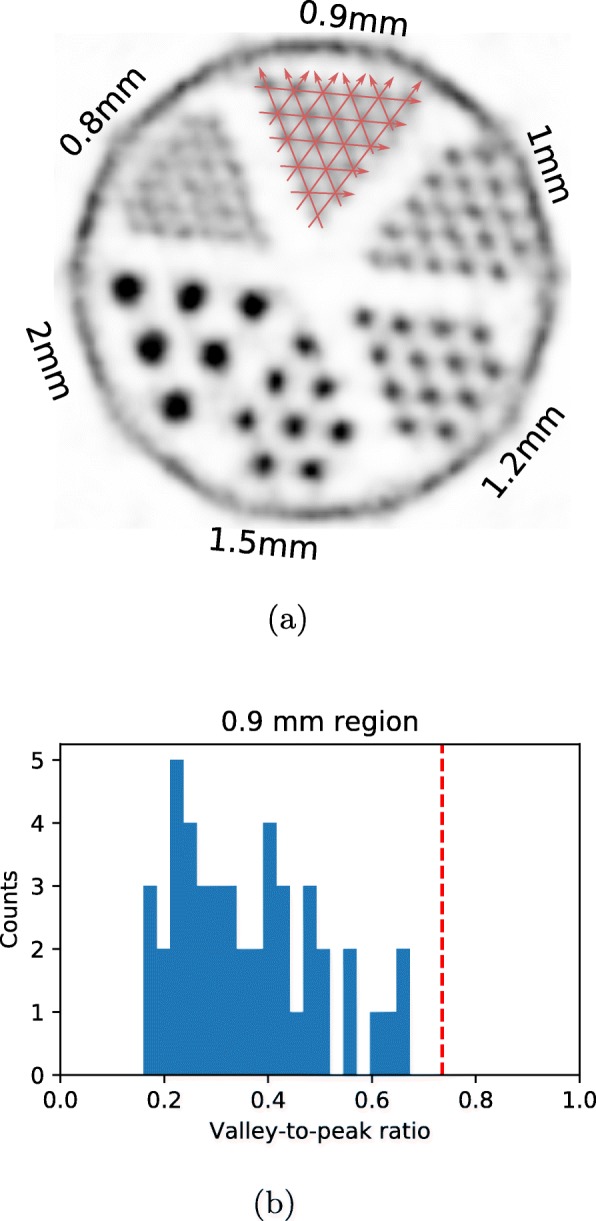
Evaluation of spatial resolution using a Derenzo phantom. **a** Reconstruction of Derenzo phantom scan. The labels indicate the diameters and the distance between the rods. The red lines show an example of profile lines which would be used for determination of valley-to-peak ratios to evaluate the spatial resolution. **b** Distribution of valley-to-peak ratios for the region with a rod distance of 0.9 mm. All ratios are below 0.735, which is marked with a red vertical line

For the scan of a Derenzo phantom, such a resolvability criterion would require to determine the valley-to-peak ratios of the profile lines over the different regions of the phantom. To include anisotropies in the spatial resolution, the profile lines should be defined over multiple angles as demonstrated in Fig. 4a. Figure 4b shows the resulting distribution of valley-to-peak ratios for the phantom’s 0.9-mm region. We would recommend that the spatial resolution is defined as the hot-rod distance in the region where at least 90% of the peak-to-valley ratios are below 0.735, i.e., the valley dips are above 26.5% for consistency with the Rayleigh criterion. Alternatively, one could define a limit based on the average peak-to-valley ratio of a region or using a different percentile than the suggested 90%. As shown in Fig. 4b, the region with distances of 0.9 mm has 100% of the valley-to-peak ratios below 0.735. For the 0.8 mm region, over half of the valley-to-peak ratios would be above 0.735 in our measurement. Thus, the resulting spatial resolution would be 0.9 mm.

To prevent arbitrary selection of peaks and valleys in a noisy reconstruction, the standard could specify a limit for the allowed deviation from the physical hot-rod distances when selecting the position of peak and valleys in the profiles of the Derenzo region.

To evaluate the influence of radial and axial offsets on the spatial resolution, the standard could specify different radial distances at which the Derenzo phantom should be placed. Similarly, the standard could also specify additional measurements of the rotated phantom to investigate the isotropy of the spatial resolution.

In our opinion, such a method would depend much less on the system’s geometry and technology and would provide a much more realistic benchmark, closely mirroring real-world use of the system. As one of the disadvantages, the precision of this method would be limited by the differences in hot-rod distances between the phantom’s region. However, with commonly used Derenzo phantoms, one would achieve a precision in the determination of the spatial resolution of 0.1 mm, which is more than adequate to assess the scanner’s viability for intended applications. Another drawback of the Derenzo phantom is that it is missing warm background activity and which could potentially lead to an artificial enhancement of spatial resolution with a high number of reconstruction iterations.

The outlined method is only intended as one possible first suggestion. We believe that developing a robust and objective method to benchmark the spatial resolution is a challenging and important research problem. One advantage of the current evaluation method is its simplicity, which simplifies Monte Carlo simulation and similar research.

As another alternative, Lodge et al. [28] have recently proposed a novel method for the measurement of clinical PET spatial resolution using a homogeneous cylinder phantom at an oblique angle. Another idea would be two use two adjacent point sources in a warm background, similar to the method described in [24].

## Scatter fraction, count losses, and random coincidence measurements

The definitions of the randoms rate, scatter rate, and scatter fraction are not satisfiable and thus ill-defined for systems employing detector material containing intrinsic radioactivity, such as LYSO or LSO scintillators, as most modern PET systems do.

To explain this issue, we give a brief summary of the NEMA standard for the measurement of the scatter fraction, count losses, and random coincidence rate in the following. The measurement is specified as a scan of an FDG-filled line source inside a scatter phantom consisting of polyethylene. The rows of the measured sinogram are centered at their maxima and the sum of all rows is calculated. In the resulting radial profile of the phantom scan, the NEMA standard specifies a signal window of 7 mm around the maximum. All event counts outside this signal window are regarded as either scatter or randoms. It is assumed that the sum of scatter and random event counts is at the same level inside the signal window as on the edges of the signal window. The sum of random and scatter event counts is denoted as *C*_*r*+*s*_, and the sum of all event counts are denoted as the total event count *C*_TOT_.

For systems without intrinsic radioactivity, the scatter fraction is supposed to be determined by assuming that the contribution of the randoms rate to the combined scatter and random counts *C*_*r*+*s*_ is negligible for measurements at a low activity. Then, the randoms rate is determined from the total event rate *R*_TOT_ and true event rate *R*_*t*_.

For systems with intrinsic radioactivity, the sum of random and scatter event counts also includes the random event counts produced by the intrinsic radioactivity and this contribution of the intrinsic randoms rate cannot be neglected at low measured activities [29]. The NEMA standard acknowledges this issue by specifying: “For systems employing detector material containing intrinsic radioactivity, the scatter fraction shall be evaluated by first evaluating the scattered event counting rate (see section 4.4.5 below).” [1, p. 13] Section 4.4.5 gives the following formula for the scattered event counting rate *R*_*s*_, which already includes the randoms rate *R*_*r*_ [1, p. 14]

1 $$  R_{s} = R_{\text{TOT}} - R_{t} - R_{r} - R_{\text{int}}  $$

The formula for the randoms rate is given above, in section 4.4.4 and it includes the scatter fraction *SF*

2 $$  {R_{r}} = R_{\text{TOT}} - \left(\frac{R_{t}}{1 - SF}\right)  $$

The scatter fraction *SF*, which is defined in the mentioned section 4.4.5, in turn includes the scattered count rate

3 $$  SF = \frac{R_{s}}{R_{t} + R_{s}}  $$

These three equations are not satisfiable for *R*_int_>0 as shown in the following proof. We insert the definition of *SF* (i.e., Eq. 3) into the definition of *R*_*r*_ (i.e., Eq. 2:

$$\begin{array}{@{}rcl@{}} {R_{r}} &= R_{\text{TOT}} - \left(\frac{R_{t}}{1 - \frac{R_{s}}{R_{t} + R_{s}}}\right) \\ &= R_{\text{TOT}} - \left(\frac{R_{t}}{\frac{R_{t} + R_{s} - R_{s}}{R_{t} + R_{s}}}\right) \\ &= R_{\text{TOT}} - R_{t} - R_{s} \end{array} $$

This is inserted into the definition of *R*_*s*_ (i.e., Eq. 1):

$$\begin{array}{@{}rcl@{}} R_{s} &= R_{\text{TOT}} - R_{t} - \left(R_{\text{TOT}} - R_{t} - R_{s}\right) - R_{\text{int}} \\ &= R_{s} - R_{\text{int}} \qquad \text{\lightning~for}\; R_{\text{int}} \neq 0 \end{array} $$

This is a contradiction, because by definition it is true that *R*_int_≠0, since the standard specifies these definitions of *R*_*r*_ and *R*_*s*_ for scanners with intrinsic radioactivity.

We can speculate on the intended meaning of the NEMA standard’s definitions. One simple explanation is that the term − *R*_int_ was simply forgotten in Eq. 2 since subtracting *R*_int_ from *R*_*r*_ would remove the contradiction. However, that would still leave the definition circular and would thus require explicit instructions on how to solve this set of equations in practice. One sensible instruction could be to neglect the influence of the randoms rate *R*_*r*_ (i.e., assume *R*_*r*_=0) in Eq. 1 for measurements at low activities to determine *R*_*s*_ and *SF*. We can then assume that *SF* is approximately constant with increasing activity and use *SF* determined at a low activity to calculate the randoms rates *R*_*r*_ and scatter rates *R*_*s*_ at higher activities.

The NEMA standard specifies the following lower activity threshold: “For scanner employing, radioactive scintillator material, measurements shall be performed until the single event rate is equal to twice intrinsic single event rate” [1, p. 11]. Our scanner has an intrinsic single event rate of 80 kcps and we reach a single event rate of 160 kcps at 430 kBq. Thus, we use this activity to estimate the scatter rate *R*_*s*_ using Eq. 1 while neglecting the randoms rate. This scatter rate is then used with Eq. 3 to determine the scatter fraction *SF*. This scatter fraction is assumed to be constant with varying activity and we use this with Eq. 2 to determine the randoms rates *R*_*r*_ at different activities. With these randoms rates we can evaluate Eqs. 1 and 3 again to determine the scatter rates and fractions at higher activities without neglecting the randoms rates.

Alternatively, the NEMA standard allows the usage of a randoms rate estimate supplied directly by the scanner. Such estimates usually use one of two techniques: one using a delayed coincidence window (DCW) [30, 31] and one based on the singles rate [30]. The singles rate (SR) method infers the randoms rate *R*_*ij*_ between to detector element *ij* from the singles rates *S*_*i*_ and *S*_*j*_ using the formula

4 $$  R^{\text{SR}}_{ij} = 2 \tau S_{i} S_{j}  $$

with the time coincidence window *τ*. However, this method systematically overestimates the randoms rate [32, 33]. Oliver et al. [34] proposed an improved method “Singles Prompt” (SP) which includes corrections based on the coincidence rate (or prompt rate) *P*_*i*_ to account for the contribution of true coincidences and pile-up events:

5 $$  R^{\text{SP}}_{ij} = \frac{2 \tau e^{- (\lambda + S) \tau}} {(1 - 2 \lambda \tau)^{2}} (S_{i} - e^{(\lambda + S) \tau} P_{i})(S_{j} - e^{(\lambda + S)\tau} P_{j}),  $$

where *λ* is the solution of the equation

6 $$ 2 \tau \lambda^{2} - \lambda + S - P\,e^{(\lambda + S) \tau} = 0.  $$

with the total singles rate $S = \sum _{i} S_{i}$ and the total prompt rate $P = \sum _{i} P_{i}$.

We have implemented these methods with the Hyperion II ^D^ scanner and can compare them empirically with the modified method the NEMA standard suggests. The NEMA standard specifies a cylindrical signal window of 8 mm around the phantom (i.e., a total diameter of 41 mm) in sinogram space. We applied an equivalent cylindrical signal window, i.e., we only determined the randoms rate for the pairs of detector elements whose line of responses intersect with the cylindrical signal window.

**Fig. 5:**
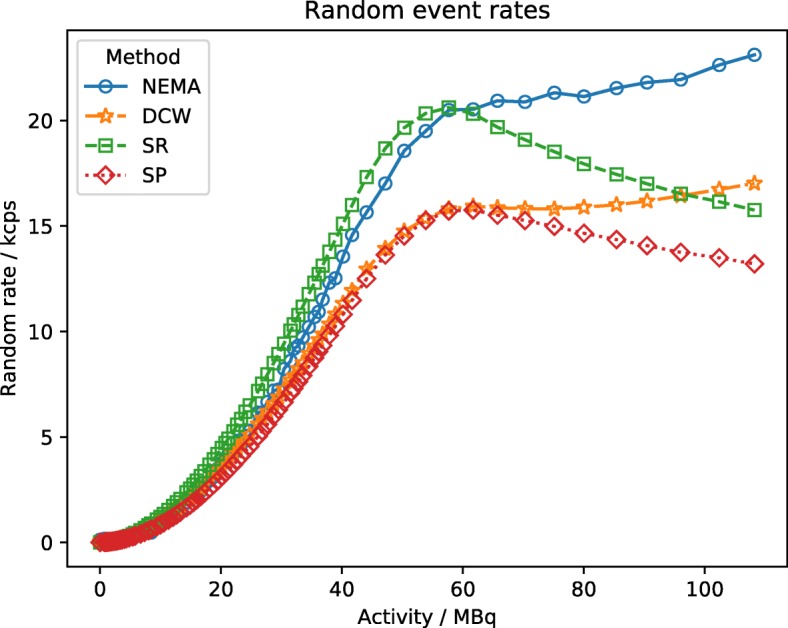
Comparison of different methods for the determination of random event rates. NEMA means a method based on the NEMA standard using Eq. 1, DCW uses a delayed coincidence window, SR is based on the singles rate using Eq. 4, and SP incorporates additional corrections using Eq. 5

Figure 5 shows the total randoms rates as a function of activity inside the scanner for the four different methods: NEMA, DCW, SR, and SP.

As expected, the randoms estimates *R*^SR^ is larger than the randoms estimate *R*^SP^: *R*^SR^≥*R*^SP^. The randoms estimate *R*^DCW^ is similar to *R*^SP^, and the modified NEMA randoms estimate *R*^NEMA^ is similar to *R*^SR^, which is known to be the less precise than *R*^DCW^ and *R*^SP^ [34].

Oliver et al. [34] showed that randoms estimates *R*^DCW^ using a delayed coincidence window (DCW) are larger or equal to the randoms estimates *R*^SP^: *R*^SR^≥*R*^DW^≥*R*^SP^. There are many publications investigating the correctness of these methods, providing evidence from theory, simulations, and measurements. For the NEMA method, on the other hand, we are not aware of any publications investigating the correctness of the method. Additionally, the verbatim definition of the NEMA method for systems with intrinsic radioactivity is contradictory, as shown above. However, we acknowledge the value of allowing a randoms estimations method which is independent on the ability to either measure delayed coincidence or single rates. Thus, one simple revision to the standard could be to correct the contradictions in the definition, possibly in the way described in this work.

All of these points apply also to the scatter rate *R*_*s*_ defined in Eq. 1 and the noise-equivalent count rate, as the definitions of these observables depend on the randoms rate.

## Sensitivity

We think the NEMA standard’s protocol for the evaluation of the sensitivity is unclear. Section 5.3 of the NEMA standard specifies to axially step a point source through the scanner. Further, Section 5.3.4 implies that a different scan for each source position should be acquired. In Section 5.4, all of the data analysis is specified for single sinogram slices *i*. For instance, the sensitivity is defined as

7 $$  S_{i} = \frac{R_{i} - R_{B,i}}{A_{\text{cal}}}  $$

with the counting rate *R*_*i*_ and the background rate *R*_*B*,*i*_ of sinogram slice *i*. However, the NEMA standard only ever references sinogram slices and never different measurements. We have one measurement per source position and each of these measurements has many sinogram slices. In other words, there are many measurements for each axial sinogram slice. Whenever the NEMA standard refers to sinogram slices, it remains unclear which measurement to consider. One possible intention could be to calculate the sum of all measurements; however, this is never explicitly stated. This would effectively create a sensitivity measurement with a virtual line source of activity *n*·*A*, where *n* is the number of measurements. Such a line source would be similar to the source distribution specified in the sensitivity protocol in the clinical NEMA NU 2-2012 standard. However, the sensitivity *S*_*i*_ is defined by the activity *A*_cal_ in Eq. 7, not a virtual activity *n* *A* of the combined measurements. Unfortunately, the NEMA standard does not define *A*_cal_ in this equation, the only definition of *A*_cal_ is in Section 1.2 as “activity at time *T*_cal_”. In conclusion, if this interpretation was the intention of the NEMA standard, multiple required instructions would be missing.

Another possible interpretation could be to take the slice *i* of the measurement where the point source is located at the center position of the slice. However, this interpretation is not consistent with the formulas given for the total system sensitivity

8 $$ S_{\text{tot}} = \sum_{\text{all}\,i} S_{i},  $$

which lack a normalization for the total number of slices. With a normalization with the total number of slices, this would effectively be an additional axial signal window around the point source, However, the size of this axial signal window would depend on the scanner’s slice thickness, giving an unfair disadvantage to high-resolution scanners. For instance, with a slice thickness of 1 mm, this axial signal window would cut into the point source. Additionally, this interpretation would not be realistic in the context of real-world applications, where the sensitivity is supposed to be an indicator of how many true coincidences one can expect for a given activity inside the scanner’s FoV.

In summary, the NEMA standard does not include any instructions on how to analyze the data of the multiple measurements it instructs to take. It only defines the sensitivity of sinogram slices without specifying the relationship of the sinogram slices and measurements with different source positions.

One consistent alternative definition of sensitivity could simply sum all sinogram slices and then divide the total coincidence counts by the acquisition time and activity for each measurement (i.e., source position). The sensitivity profile would consequently be defined as this total sensitivity as a function of the source position. To calculate the mouse- and rat-equivalent sensitivities, one would average this sensitivity profile inside the central 7 cm or 15 cm. Because the NEMA standard specifies a transversal signal window with a width of 20 mm in sinogram space, it would be consistent to apply the same signal window around the point source in axial direction. We believe that this method is already used in multiple performance evaluations based on NEMA [5, 12, 14, 35], although the exact details of the methods are usually not explained.

Therefore, the ambiguity of the NEMA standard can lead to unclear and incomparable results in performance publication based on NEMA, impeding an objective comparison of different sensitivity results.

For instance, Prasad et al. [13] seem to follow the formulas given by NEMA quite closely, without clearly specifying how the data of the different measurements at different source measurement is used in the data analysis. The reported sensitivity profile has data points above 1 cps/Bq, i.e., an impossible sensitivity larger than 100% for the central slices. They claim a total absolute sensitivity of 12.74%, which is implausibly large compared to the expected geometric sensitivity of 12.9%. We calculated this ideal geometric sensitivity using their scanner’s diameter, axial length, and crystal thicknesses with the simple geometric model explained in [4]. The usual ratio between measured peak sensitivity and geometric sensitivity is between 0.3 and 0.5 [4].

## Image quality, accuracy of attenuation, and scatter corrections

The NEMA standard defines several observables for quantitative analysis of the image quality phantom. The uniformity is defined as the relative standard deviation of all voxels in a large cylindrical volume of interest over the uniform region in the image quality phantom. For determination of the recovery coefficients, the image slices along the central 10 mm of the hot rods are averaged. Then, the recovery coefficients are defined as the maximum values in a circular region of interest around the hot rods with different diameters, divided by the mean activity in the volume of interest over the uniform region. The issue with this definition is that the recovery coefficients are correlated with the uniformity: The maximum value of a randomly distributed sample increases with variance, even if the mean value of the distribution is constant. Thus, this definition of the recovery coefficients does not measure the mean recovery in the hot rods, but measures a combination of recovery and variance. With a high variance and a good recovery the recovery coefficients can even reach values larger than 1.

**Fig. 6:**
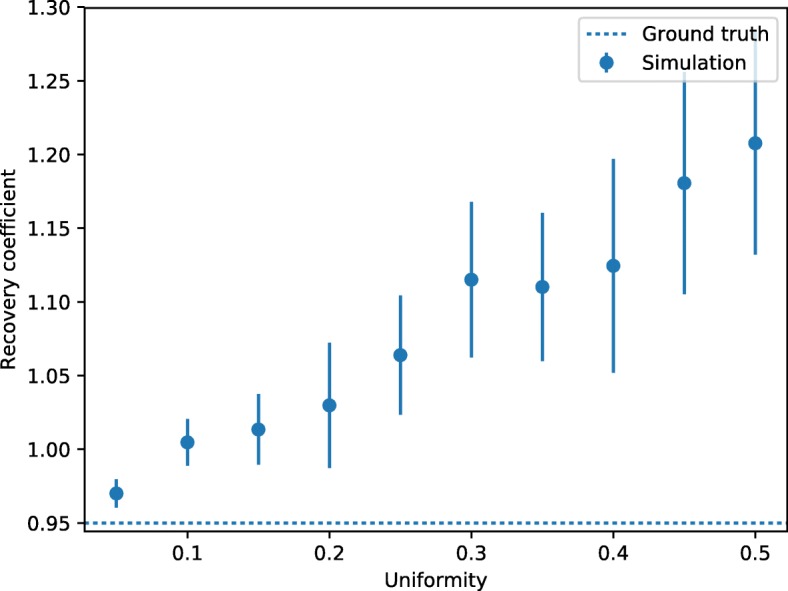
Simulated recovery coefficient of the 5-mm rod as a function of the uniformity. The ground truth for the recovery was 0.95. The simulated recovery coefficients are always larger than the ground truth and increase with increasing variance (i.e., larger uniformity values)

We can demonstrate this behavior in a simple Monte Carlo simulation, where we assume that the reconstructed activity in a voxel follows a normal distribution with the standard deviation given by the uniformity. The simulated geometry is the NEMA image quality phantom. Figure 6 shows the simulated recovery coefficients of the 5-mm rod as a function of the uniformity. The ground truth for the recovery coefficient for the activity in the rod was 0.95. The data analysis follows the NEMA standard, i.e., the recovery coefficient is defined by the maximum activity in the region of interest. The drawn errors are calculated from the errors on the mean of the averaged pixels in the region of interest. The simulation demonstrates that the recovery coefficient is always overestimated compared to the ground truth and increases with increasing variance (i.e., larger uniformity values).

**Fig. 7:**
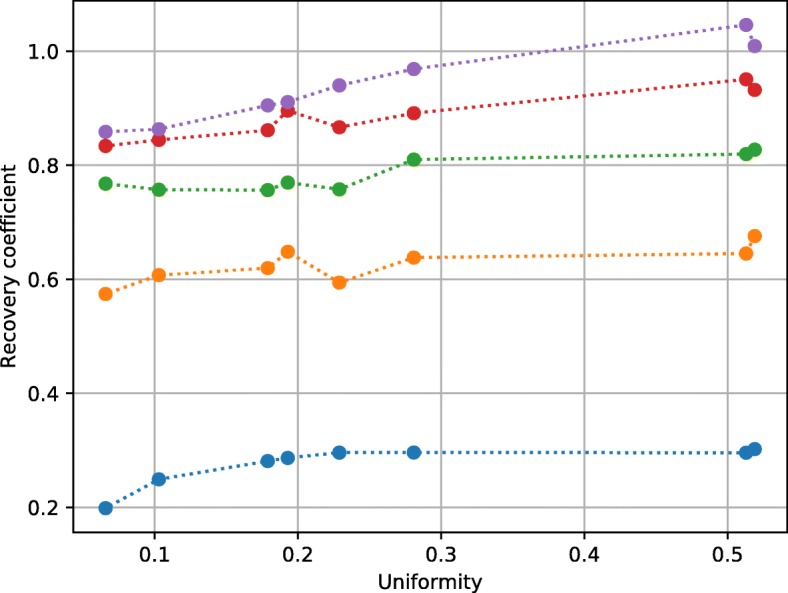
Measured recovery coefficients as a function of uniformity. The curves of the recovery coefficients correspond to rods with diameters of 5 to 1 mm, from top to bottom. Each different uniformity value corresponds to a different filter width used during reconstruction. A larger filter reduces variance and therefore increases uniformity (i.e., decreasing relative standard deviation). The recovery coefficients are increasing with increasing uniformity values, so overall image quality performance is a trade-off between uniformity and recovery

Thus, the NEMA standard’s definition of the recovery coefficients hampers an easy comparison of different scanner’s recovery performance, because the recovery and uniformity must be compared at the same time. In other words, the same scanner can achieve different recovery performance at different uniformity points. The user can influence the uniformity with parameters such as the amount of filtering during reconstruction. Figure 7 shows measured recovery coefficients as a function of varying uniformity. Each uniformity value corresponds to different widths of a Gaussian kernel used during reconstruction of a scan of the image quality phantom. We used the maximum likelihood expectation maximization reconstruction described in [36]. As predicted by the Monte Carlo simulation, the recovery coefficients are correlated with the relative standard deviation in the uniformity region: Both values decrease with large filter width, i.e., reduced variance in the image. Of course, it is not unexpected that the recovery decreases with stronger filtering during reconstruction. However, the observed effect is on top of the expected decrease in recovery due to filtering. Using the NEMA standard’s observables, improving the uniformity performance will always lead to a loss in observed recovery, regardless of whether the actual true recovery degraded or not. When conducting a NEMA performance evaluation, one has to chose an arbitrary point on the uniformity and recovery curve resulting in one of many possible results, which are difficult to compare with the results of other scanners.

As another minor issue, the NEMA standard derives the standard deviation of the recovery coefficients from the standard deviations of the line profiles along axial directions and the standard deviations of the uniform regions using Gaussian error propagation. This is not the correct standard deviation of the recovery coefficient, because the standard deviation of the maximum value of a randomly distributed value is not the standard deviation of the underlying distribution.

Fixing the definition of the recovery coefficients is not trivial. The NEMA standard probably uses the maximum due to the small diameters of the hot rods. For the very small rods, very few, if any, voxels lie clearly in the center of the rods. Alternative definitions using the mean in a volume of interest will therefore be biased by the smaller reconstructed activity in the border regions of the rods. However, with today’s high-resolution PET scanners, we believe it would be possible for most scanners to define volume of interest (VoI) inside the hot rods and then define the recovery coefficients using the mean reconstructed activity inside the VoI. Even if these VoIs would partially include the border regions of the rods, it would still at least be a comparable measure of recovery for every scanner. For the larger rods it should not be any problem to define VoI which are well inside the hot rods with a sufficient number of voxels. It is these larger rods where the current definition of recovery coefficients leads to basically a recovery of 1 or larger for all current scanners, hindering a differentiation of subtle differences in recovery between the scanners.

Another addition to the NEMA standard could be a scan of the image quality phantom at low activities to evaluate the performance of the reconstruction under low statistics, because iterative reconstruction methods usually exhibit bias at low statistics [37, 38]

Another research opportunity would be the development of a new phantom geometry using hot small spheres instead of axial hot rods. Such a geometry would be more similar to hot lesions in rodents and thus provide a benchmark of contrast recovery which is more similar to uptake in rodents. It would also be better comparable to the phantom used in the clinical NEMA NU-2 standard [39]. Ideally, such hot spheres would be situated in a warm background, although that would introduce the problem of cold sphere walls [40]. However, manufacturing a practical phantom with millimeter-sized fillable spheres is mechanically challenging.

## General points

The NEMA standard does not explicitly mandate the use of the same settings for each measurement. Most scanners offer a multitude of settings for measurements and data processing, such as trigger settings, coincidence, and energy window sizes and quality filters for gamma interactions (e.g., detector scatter rejection [41, 42]). The choice of setting parameters requires often a trade-off for different performance parameters. For example, the sensitivity benefits from wide energy and coincidence windows and no quality filters, while the image quality and spatial resolution benefit from narrow windows and strict quality filters. One could report very misleading performance results by optimizing the settings for each performance measurement separately, thus achieving performance results which are unattainable at the same time in real-world applications.

While following the standard, many performance publications based on NEMA do neither state if they used the same settings for every measurement explicitly nor report all used settings for each measurement. For example, Nagy et al. [5] use wide energy windows for the sensitivity and count rates measurements and a narrow energy window for the measurement of spatial resolution. They do not report any settings for the image quality measurement.

Another issue is the mandated use of sinograms. The data analysis for every measurement except the image quality measurement are described on sinograms. However, most modern scanners store their data in listmode format and might only implement sinogram support to conduct the NEMA measurements. To our knowledge, all NEMA NU-8 measurements published in the last 5 years used listmode files for data acquisition and had to convert the listmode files to sinograms after the measurements [4–15, 43]. Spinks et al. [8] even mentions that the calculation of scatter fractions were omitted due to missing sinogram support, so this performance evaluation did apparently only use listmode data for the data analysis. The number of scintillator crystals is usually above 30 000 in modern small-animal PET systems, so that full 3D sinograms have a file size of multiple gigabytes even for very short measurements. Listmode files on the other hand are usually much smaller, making sinograms much more unwieldy.

PET scanners with monolithic scintillator blocks [15, 44] might not have clear bins which correspond to sinogram bins. For instance, such detectors might use continuous regression methods for determining the most likely position of gamma interactions [45].

The data analyses in the NEMA standard could be specified without the use of sinograms, since most of the specified cuts in the sinograms could be specified as cylindrical cuts in the scanner’s field of view. The standard could still allow the use of sinograms as one possibility to implement the specified geometric cuts for backwards compatibility.

## Conclusion

Eleven years after the publication of the NEMA NU-4 standard, we believe it is time for a revision of the standard. In this work, we have pointed out several flaws in the standard which should be addressed in the next version. Additionally, the new technological developments in the last decade would warrant discussing an updated version in itself. With this publication, we would like to open this discution.

## Data Availability

The datasets used and/or analyzed during the current study are available from the corresponding author on reasonable request.
